# Interobserver variability of injury severity assessment in polytrauma patients: does the anatomical region play a role?

**DOI:** 10.1186/s40001-021-00506-w

**Published:** 2021-04-15

**Authors:** Eftychios Bolierakis, Sylvia Schick, Kai Sprengel, Kai Oliver Jensen, Frank Hildebrand, Hans-Christoph Pape, Roman Pfeifer

**Affiliations:** 1 Department of Trauma and Reconstructive Surgery, University Hospital RWTH Aachen, RWTH University of Aachen, Pauwelsstr. 30, 52074 Aachen, Germany; 2 Institute of Legal Medicine, Ludwig Maximilians Universitaet LMU, University of Munich, Munich, Germany; 3 Department of Trauma, University Hospital Zurich, University of Zurich, Zurich, Switzerland

**Keywords:** Trauma, Injury severity, ISS, AIS, NISS, Interobserver variability

## Abstract

**Background:**

The Abbreviated Injury Scale (AIS) and Injury Severity Score (ISS) are widely used to assess trauma patients. In this study, the interobserver variability of the injury severity assessment for severely injured patients was analyzed based on different injured anatomical regions, and the various demographic backgrounds of the observers.

**Methods:**

A standardized questionnaire was presented to surgical experts and participants of clinical polytrauma courses. It contained medical information and initial X-rays/CT-scans of 10 cases of severely injured patients. Participants estimated the severity of each injury based on the AIS. Interobserver variability for the AIS, ISS, and New Injury Severity Score (NISS) was calculated by employing the statistical method of Krippendorff's α coefficient.

**Results:**

Overall, 54 participants were included. The major contributing medical specialties were orthopedic trauma surgery (*N* = 36, 67%) and general surgery (*N* = 13, 24%). The measured interobserver variability in the assessment of the overall injury severity was high (α _ISS_: 0.33 / α _NISS_: 0.23). Moreover, there were differences in the interobserver variability of the maximum AIS (MAIS) depending on the anatomical region: α_head and neck_: 0.06, α_thorax_: 0.45, α_abdomen_: 0.27 and α_extremities_: 0.55.

**Conclusions:**

Interobserver agreement concerning injury severity assessment appears to be low among clinicians. We also noted marked differences in variability according to injury anatomy. The study shows that the assessment of injury severity is also highly variable between experts in the field. This implies the need for appropriate education to improve the accuracy of trauma evaluation in the respective trauma registries.

**Supplementary Information:**

The online version contains supplementary material available at 10.1186/s40001-021-00506-w.

## Background

Polytrauma continues to be one of the leading causes of mortality, especially for persons under the age of 45, and has socioeconomic implications despite modern developments in acute medical care and prevention [1, 2]. Accurate identification of these patients and consistent grading of the respective injury patterns play a pivotal role in hospital quality benchmarking, allocation of resources, and data comparability between different trauma centers and countries [3–6].

The assessment of injury severity of polytraumatized patients is mainly based on the use of standardized anatomical-based coding employing the Abbreviated Injury Scale (AIS) and Injury Severity Score (ISS), as well as the New Injury Severity Score (NISS) [7–9]. Following the introduction of its first version in 1969 [10], the AIS has gone through validation processes with multiple updates, the latest in 2015. Since the early 1990s, the AIS has become an integral part of the anatomical definitions of polytrauma [11–16], which were established as an attempt to create more specificity than the older descriptions by Border et al. (1975), Faist et al. (1983), and Tscherne et al. (1986) [17–19]. While being primarily created for communication between medical and nonclinical investigators, the AIS and consequently the ISS are currently considered the ‘gold standard’ of injury severity assessment in trauma registries worldwide [20–25]. Nevertheless, issues concerning its high interobserver variability and subjectivity were recognized early on [26–28].

Injury assessment according to the AIS is being taught today within the scope of respective courses for the purpose of providing dedicated coding specialists. Discussing their results in the context of current literature, Maduz et al. suggested there was a negative influence of the coder’s medical experience on the accurate assessment of the injury severity through the AIS grading system [22]. This assumption contradicts the observations of the primary analysis on the subject from MacKenzie et al., whose research supported the hypothesis that medical personnel fare better than non-medical technicians [27]. The chronological gap between these statements could imply the confounding role of the trauma system evolution or the newer versions of the AIS grading system. To our experience, coding is often not conducted by specially trained medical personnel and clinicians with varying coding experience, and it is primarily based on evaluating the patient charts after their discharge. This raises the question of how accurately clinicians nowadays, who did not participate in respective educational programs but actively take part in the everyday medical care of injured individuals, evaluate injury severity in the context of this coding system.

Therefore, the aim of the current study is to measure the interobserver variability in the assessment of injury severity among medical clinicians interested in trauma management from around the world. The influence of the demographic backgrounds of the surveyed clinicians is also being investigated, with special focus on the different injured anatomical regions. We hypothesized that injury severity assessment is highly variable between observers, with values depending on the respective anatomical injury pattern.

## Methods

### Ethical considerations

The study protocol was approved by the local ethics committee (Ethics Committee at the RWTH Aachen Faculty of Medicine, EudraCT-EK 005/17), and there was compliance with the principles of the seventh revision of the Declaration of Helsinki, as well as the Good Clinical Practice Guidelines throughout the study.

### Questionnaire

The study design is a questionnaire-based self-reported survey. Following the paradigm of expert assessment of injury cases from previous interobserver variability studies, a questionnaire was created with a description of 10 cases of polytraumatized patients, including X-ray examinations, information about trauma mechanisms, injuries in different anatomical regions, and various pathophysiological parameters [27, 29–31]. The questionnaire also included questions about the surveyed participants’ demographic and occupational background, e.g., specialty, gender, level of medical training, years of working experience, frequency of treatment of polytraumatized patients (cases/month), level of clinical trauma care (1–5 according to the American Trauma Society), country of medical education, and country of current employment [32]. The anatomical injuries were sub-grouped according to their respective ISS body regions: head and neck, face, thorax (including thoracic spine), abdomen (including visceral pelvis/lumbar spine), extremities (including osseous pelvis/shoulder girdle), and external (including skin/soft tissues) [8]. The overall maximum AIS (MAIS) of each body region, ISS, and NISS were implemented as expressions of the overall injury severity, which was calculated based on the participants’ AIS estimates [7–9]. The X-ray material originated from the radiological database of the Department of Orthopedic Trauma, RWTH Aachen University, Aachen, Germany. The presented patient cases were fictively conceptualized based on real injury patterns and trauma mechanisms, making identification of a real individual patient impossible. The respective frequency of the chosen injuries was based on the yearly report of the national trauma registry of Germany (TraumaRegister DGU®). Each injury pattern was preliminarily assessed by an Association for the Advancement of Automotive Medicine (AAAM) certified specialist for the purposes of expert calculation of the respective AIS, ISS, and NISS (Additional file 1: Table S1). According to this assessment, the presented patient cases had a median ISS of 34 (IQR 29–38) and a median NISS of 41 (IQR 33–54) (Table 1) [7–9].

**Table 1:** Overview of the overall number of codes and median AIS per ISS-anatomical region of the presented polytrauma cases

	Head and neck^a^	Face^a^	Thorax (including thoracic spine)^a^	Abdomen (including visceral pelvis/lumbar spine)^a^	Extremities (including osseous pelvis/shoulder girdle)^a^	External (including skin/soft tissues)^a^
Number of codes	18	3	24	8	22	6
Median AIS (IQR)	3 (2–4)	2 (1–2)	3 (2–4)	2 (2–3)	2 (2–3)	1 (1–1)

Codes were allocated from an AAAM-certified specialist

AIS: Abbreviated Injury Scale; ISS: Injury Severity Score; AAAM: Association for the Advancement of Automotive Medicine; IQR: interquartile range

^a^ISS anatomical region

### Study population

The questionnaire was distributed within the frame of trauma courses of international traumatological congresses (Cooperative Course: Polytrauma Management Beyond ATLS, https://polytraumacourse.com). These interdisciplinary trauma courses are addressed to general surgeons, neurosurgeons, orthopedic trauma surgeons, intensive care, and emergency physicians. Furthermore, this course discusses the entire clinical course of a polytraumatized patient; from preclinical treatment until rehabilitation. The surveyed clinicians were asked to estimate the injury severity of the various anatomical regions as well as the entire injury severity in the form of the AIS [7]. No AIS dictionary or similar conversion tool was used during the supervised assessment of the questionnaire. Therefore, the respective data entry is to be evaluated as an estimate and not as AAAM-certified coding.

### Statistical analysis

The collected data were stored on an Excel spreadsheet (Excel 2013, Microsoft Corp., Redmond, WA, USA). The MAIS of the respective anatomical region was used to examine the influence of the injury anatomy on the observed interobserver variability. Categorical values were expressed as frequencies/percentages, while median and interquartile range (IQR) values were used for continuous variables and 95% confidence intervals (95% CI) were reported. The interobserver variability was measured utilizing the statistical method of Krippendorff’s alpha (α) reliability coefficient [33]. The main advantage of this statistical analysis over the more popular kappa statistics and intraclass correlation coefficient is its extended robust capability to measure the interobserver variability irrespective of sample size, multiple (more than 2) coders or missing data. All measurement levels can be tested. Krippendorff’s alpha reliability coefficient can also produce negative values when coders systematically agree to disagree, meaning that the coders are doing worse than by chance alone and indicating that at least some structural differences exist [34]. Missing values were excluded with pairwise deletion, and the respective numerical results were rounded to two decimal places. With the value “0” representing total disagreement and the value “1” representing perfect agreement among the participants, we used Fleiss’s guidelines on kappa interrater reliability statistics as a basis for interpretation [35]: > 0.75 (excellent agreement beyond chance), 0.40–0.75 (fair-to-good agreement beyond chance), and < 0.40 (poor agreement beyond chance). The statistical analyses were conducted with SPSS (IBM Corp. released 2017. IBM SPSS Statistics for Windows, Version 25.0. Armonk, NY: IBM Corp.).

## Results

### Demographic parameters

Overall, 54 questionnaires with data from participants (47 male, 7 female) with various levels of medical education (20 residents; 15 attending specialists; 9 consultants; 10 heads of departments/professors) were included in the study. According to the descriptive analysis of their demographic backgrounds, the participants received medical education in 23 different countries (regional frequency: Europe (*N* = 26, 48%), Asia (*N* = 17, 32%), and Africa (*N* = 11, 20%)). The main contributing specialties were orthopedic trauma surgery and general surgery, with 67% (*N* = 36) and 24% (*N* = 13) of the surveyed population, respectively. There were also one pediatric surgeon, one anesthesiologist, one medical intensive care specialist, and two participants who did not state their medical field of expertise. Each level of institutional trauma care was represented in the study: Levels 1–2: 57% (*N* = 31), Level 3: 13% (*N* = 7), and Levels 4–5: 30% (*N* = 16). The median working experience of the participants was 10 years (IQR 5–20), and they were treating a median of three polytrauma patients every month (IQR 2–10) (Table 2). Their overall assessment of the presented injury cases resulted in a median ISS of 38 (IQR 29–54) and a median NISS of 48 (IQR 34–66). They correctly estimated 32% of the depicted injuries (Table 3).

**Table 2:** Demographic and occupational background of the participants

Demographic data	*N*
Overall	54
Gender	
Male	47
Female	7
Specialty	
General surgery	13
Orthopedic trauma surgery	36
Other	5
Level of medical education	
Resident	20
Attending specialist	15
Consultant	9
Head of the department/professor	10
Working experience in years	
0–2	6
3–6	16
7–12	7
13–20	8
> 20	17
Working experience in polytrauma-cases per month	
0	3
1	8
2–3	17
4–5	6
6–10	10
> 10	10
Level of medical trauma care	
1–2	31
3	7
4–5	16
Region of medical education	
Europe	26
Asia	17
Africa	11
Region of current employment	
Europe	33
Asia	10
Africa	11

**Table 3:** Overview of the total number of correctly assessed injuries and median MAIS per ISS-anatomical region of the presented polytrauma cases as estimated by the study participants

	Head and neck^a^	Face^a^	Thorax (including thoracic spine)^a^	Abdomen (including visceral pelvis/lumbar spine)^a^	Extremities (including osseous pelvis/shoulder girdle)^a^	External (including skin/soft tissues)^a^
Total number of assessed injuries	972	162	1296	432	1188	324
Number of correctly estimated injuries (%)	231 (24)	58 (36)	368 (28)	102 (24)	474 (40)	147 (45)
Median MAIS (IQR)	4 (4–5)	2 (2–3)	4 (3–5)	3 (3–5)	3 (2–4)	1 (1–2)

MAIS, Maximum Abbreviated Injury Scale; ISS, Injury Severity Score; IQR, interquartile range

^a^ ISS anatomical region

### Interobserver variability

The overall assessment of the injury severity was highly variable, indicating poor agreement, among the observers, as indicated by the respective α scores of the general surveyed population (α_ISS_: 0.33, 95% CI 0.23–0.42; α_NISS_: 0.23, 95% CI 0.12–0.34; and α_MAIS_: 0.17, 95% CI 0.09–0.25). While there were differences in the assessment of the overall injury severity among the various demographic subgroups, our results did not demonstrate a statistically significant influence pattern of the level of medical education, the working experience, or the region of the participants on the measured interobserver variability, as suggested by the random overlap of the respective confidence intervals (Table 4).

**Table 4:** Interobserver variability of ISS, NISS and MAIS

Observers	ISS (95% CI)	NISS (95% CI)	MAIS (95% CI)
Overall	0.33 (0.23–0.42)	0.23 (0.12–0.34)	0.17 (0.09–0.25)
Level of medical education			
Resident	0.38 (0.29–0.47)	0.19 (0.07–0.31)	0.13 (0.04–0.20)
Attending specialist	0.26 (0.15–0.35)	0.16 (0.04–0.28)	0.19 (0.11–0.28)
Consultant	0.15 (0.03–0.27)	0.15 (0.01–0.29)	0.08 ((− 0.05)–0.21)
Head of the department/professor	0.43 (0.33–0.52)	0.40 (0.31–0.48)	0.27 (0.18–0.37)
Working experience in years			
0–2	0.55 (0.39–0.69)	0.54 (0.35–0.71)	0.26 (0.09–0.42)
3–6	0.30 (0.21–0.39)	0.17 (0.05–0.29)	0.11 (0.02–0.20)
7–12	0.30 (0.14–0.45)	0.17 ((− 0.01)–0.32)	0.12 ((− 0.05)–0.29)
13–20	0.11 ((− 0.03)–0.25)	0.17 ((− 0.01)–0.33)	0.15 (0.01–0.29)
> 20	0.35 (0.25–0.44)	0.22 (0.11–0.33)	0.18 (0.09–0.27)
Working experience in polytrauma-cases per month			
0	0.50 (0.21–0.76)	0.51 (0.20–0.78)	0.25 (0.04–0.47)
1	0.34 (0.22–0.46)	0.40 (0.28–0.51)	0.22 (0.10–0.35)
2–3	0.27 (0.17–0.38)	0.22 (0.10–0.34)	0.22 (0.13–0.30)
4–5	0.04 ((− 0.13)–0.20)	0.02 ((− 0.17)–0.19)	− 0.04 ((− 0.22)–0.13)
6–10	0.44 (0.34–0.53)	0.35 (0.22–0.46)	0.23 (0.12–0.33)
> 10	0.41 (0.33–0.50)	0.04 ((− 0.09)–0.16)	0.01 ((− 0.11)–0.12)
Level of medical trauma care			
1–2	0.39 (0.29–0.47)	0.22 (0.11–0.34)	0.18 (0.08–0.28)
3	0.22 (0.04–0.38)	0.20 (0.03–0.37)	0.16 (0.01–0.30)
4–5	0.33 (0.23–0.43)	0.26 (0.13–0.38)	0.19 (0.10–0.28)
Region of medical education			
Europe	0.29 (0.19–0.40)	0.16 (0.03–0.28)	0.13 (0.04–0.22)
Asia	0.36 (0.28–0.44)	0.37 (0.25–0.47)	0.28 (0.20–0.36)
Africa	0.44 (0.34–0.54)	0.23 (0.12–0.33)	0.26 (0.16–0.36)
Region of current employment			
Europe	0.33 (0.23–0.43)	0.21 (0.08–0.33)	0.14 (0.05–0.22)
Asia	0.25 (0.16–0.34)	0.27 (0.17–0.37)	0.15 (0.05–0.25)
Africa	0.45 (0.35–0.55)	0.23 (0.12–0.34)	0.26 (0.16–0.36)

Statistical method of Krippendorff’s alpha (α) reliability coefficient: > 0.75 (excellent agreement beyond chance), 0.40–0.75 (fair-to-good agreement beyond chance) and < 0.40 (poor agreement beyond chance)

ISS: Injury Severity Score; NISS: New Injury Severity Score; MAIS: Maximum Abbreviated Injury Scale; CI: confidence interval

Considering the various ISS anatomical regions, there were marked differences in interobserver agreement (Table 5). While the overall interobserver variability was high, indicating poor agreement, for the head and neck, face, and external regions (α_head and neck_: 0.06, α_face_: 0 and α_external_: 0.06), the surveyed participants showed fair-to-good agreement on evaluating injuries to the thorax and extremities (α_thorax_: 0.45, α_extremities_: 0.55). The specialties of the participants seemed to be a contributing factor. Orthopedic trauma surgeons demonstrated fair-to-good agreement (α: 0.59, 95% CI 0.54–0.64) when assessing injuries of the extremities. At the same time, general surgeons showed markedly lower interobserver variability (α: 0.44, 95% CI 0.37–0.52) compared with the entire surveyed population (α: 0.27, 95% CI 0.20–0.33) regarding the abdominal region.

**Table 5:** Interobserver variability of MAIS according to ISS-anatomical region

Observers	Head and neck^a^	Face^a^	Thorax (including thoracic spine)^a^	Abdomen (including visceral pelvis/lumbar spine)^a^	Extremities (including osseous pelvis/shoulder girdle)^a^	External (including skin/soft tissues)^a^
Overall	0.06	0	0.45	0.27	0.55	0.06
Specialty						
General surgery	0.05	− 0.03	0.47	0.44	0.46	− 0.02
Orthopedic trauma surgery	0.04	0	0.45	0.21	0.59	0.07

Statistical method of Krippendorff’s alpha (α) reliability coefficient: > 0.75 (excellent agreement beyond chance), 0.40–0.75 (fair-to-good agreement beyond chance) and < 0.40 (poor agreement beyond chance)

MAIS: Maximum Abbreviated Injury Scale; ISS: Injury Severity Score

^a^ ISS anatomical region

## Discussion

The accurate recognition and evaluation of polytraumatized patients is a main prerequisite of current traumatological research. Therefore, grading systems are required with a high level of agreement between experts in the field [22, 36, 37]. The presented study confirmed our primary hypothesis and revealed the following results:

1. The assessment of the injury severity of polytraumatized patients among surgical experts varied widely, and;
2. the variation depended considerably on the various injured anatomical regions (fair-to-good interobserver agreement: anatomical region of thorax (incl. thoracic spine) and extremity (incl. osseous pelvis/shoulder girdle), poor interobserver agreement: anatomical regions of head and neck, face, abdomen (incl. visceral pelvis/lumbar spine), and external (incl. skin/soft tissues)). This could also imply the influencing role of the coder’s medical field of expertise.

The highly variable assessment of injury severity among surgical experts delineates the possible influence of respective individual traits as well as the complexity of the current coding system. Discrepancies in injury coding, indicating over- or underestimation of the injury severity, between clinicians can result in relevant differences in therapeutic decisions over the treatment course of polytraumatized patients. Furthermore, direct comparability of research data from different institutions is restricted when it comes to developing novel polytrauma management systems. Therefore, specially trained coding specialists are still needed to ensure the reliability of quality hospital benchmarking, accurate documentation in the various polytrauma registries, and consequently, the comparability of studies in this field. Our results confirmed the variability issues of the AIS and injury severity scoring reported in previous studies conducted by McKenzie et al. and Zoltie et al. [26, 27, 38]. The Zoltie et al. study found that for 16 patients assessed by 15 observers, there was a 28% probability of 2 observers agreeing on the same score [38], almost reflecting the results of our study (α_ISS_: 0.33, α_NISS_: 0.23). Maduz et al. regarded the inconsistent ISS-AIS cut-off values as a pivotal influential factor in accurate polytrauma identification, despite reporting excellent interrater agreement for the AIS and ISS utilizing the intraclass correlation coefficient (ICC) on three specially trained observers [22]. On the contrary, Ringdal et al. questioned the reliability of the AIS-based ISS-NISS [30]. In that study, 10 Norwegian AAAM-certified trauma registry AIS coders evaluated 50 cases of polytraumatized patients. ICC was again used to measure the interobserver reliability, resulting in fair interrater agreement for both the ISS and NISS (ICC: 0.51). The observer’s experience in coding did not seem to significantly influence the results. While the ISS anatomical regions were used for descriptive statistics, there was no assessment of the respective interobserver variability or analysis of the observers’ demographic backgrounds.

Investigating the AIS coding in the Queensland Trauma Registry, Neale et al. [31], despite recording a high variability in AIS estimates (39% probability of agreement between two observers), found excellent interrater reliability for the ISS (ICC: 0.9), which disagrees with the results of our study. For the purposes of the Neale et al. study, six specially educated coders assessed 120 injury cases. The high interobserver variability of the AIS-based definitions of a polytraumatized patient was confirmed by a recent study by Pothmann et al. [39]. In their study, two observer groups coded a total of 187 polytrauma cases. One observer group consisted of a doctoral student, while the coding for the second observer group was conducted by four interns with at least 3 years of clinical experience. The dependence of the interobserver variability on anatomical region or on the demographic characteristics of the observers was not a subject of investigation in this study. Furthermore, the focus was mainly on the different cut-off values of the various polytrauma definitions, therefore only indirectly assessing the interrater variability of the current injury severity coding systems. Discussing the results, Pothmann et al. advocated the comparatively greater interobserver agreement on polytrauma identification based on MAIS, which partly confirms the respective results on pediatric trauma from Brown et al. [39, 40]. This could also imply the influence of the injured anatomical regions on the measured interobserver variability.

While most of the interobserver studies on this subject to date have mainly attempted to define polytrauma, there has been little evidence found concerning the direct interobserver variability of injury severity assessments depending on different anatomical regions or injury patterns. There is also scarce information regarding the influence of the different demographic characteristics of the surveyed observers. The current study attempts to focus on these issues by including more participants than similar studies and supports the argument that there is no standardized perception of trauma magnitude among surgical specialists from around the world.

The scientific literature provides limited analyses of the effect of raters’ experiences or training, but there is a definite pattern to be recognized. Waydhas et al. observed a significant deviation of measured trauma scores based on different professions and education [41]. Clinicians fared slightly better than non-clinicians in the study of MacKenzie et al. (1985), and Josse et al. supported the role of training in improving agreement in injury coding [27, 42].

The high overall interobserver variability among coders/specialists who were not specially trained supports the belief that specific education is necessary to improve the quality of injury severity assessment in polytraumas. Moreover, we observed distinct differences based on the injured anatomical region and the main specialty field of the participants. The measured interobserver variability was lower in anatomical regions with higher incidences of involvement in polytraumatized patients, such as the thorax and the extremities. In this context, the influencing role of familiarity with respective injury patterns as well as the differing complexity of assessment depending on the anatomical region could be implied. The lower interrater reliability in the ISS regions of the head and face, despite their high incidence in severe trauma, could be explained by the lack of neurosurgeons and maxillofacial surgeons in the surveyed population. At the same time, general surgeons showed higher interobserver agreement on assessing abdominal injuries, while orthopedic traumatologists could reach fair-to-good agreement on extremity injury patterns, further suggesting the influence of the respective working field. Furthermore, while the injuries of the thorax or the extremities show a repetitive simple pattern, assessment of head injuries underlies severity variation that is not always apparent.

### Limitations and strengths

Employing a questionnaire in paper form with considerable time needed for completion and the multiplicity of its requirements led to a restricted number of participants, different medical specialties and presented cases (10 polytrauma patients) with possible influence of the respective variation on the measured interobserver variability. Another study limitation was the assessment of the injuries based on written descriptions or small-sized depictions of conventional X-ray and CT examinations, rather than on modern radiography image processing. Manual or electronic tools as a reference guide for AIS coding were not provided. Studies with more simplified layouts based on online digital formats could be the solution to these limitations, enabling the inclusion of more participants and expanding their demographic or occupational backgrounds.

Nevertheless, our study also demonstrated certain strengths. We included 54 participants, thus forming an international cohort of surgical experts with various demographic characteristics. Utilizing Krippendorff’s alpha (α) reliability coefficient, we were able to analyze the interobserver variability results according to patients’ different injured anatomical regions or the demographic backgrounds of the observers in order to understand the factors influencing the injury assessment. The questionnaire was processed under defined conditions (Cooperative Course: Polytrauma Management Beyond ATLS).

## Conclusions

This study is one of the first documented efforts to quantify interobserver variability in the assessment of injury severity of polytraumatized patients based on different injured anatomical regions, and the demographic backgrounds of medical specialists who participate in trauma care. The high observed interrater variability among experts in the field strengthens the call for appropriate education to improve the accuracy of trauma evaluation in the respective trauma registries and set the basis for efficient hospital benchmarking. It indicates the importance of interdisciplinary training of trauma specialists, and hints at the limitations of the AIS as a freehand guide for estimating injury severity. Future studies with more participants are needed to further investigate the influencing role of the demographic background of the practicing clinicians on the respective interobserver variability.

## Supplementary Information

**Additional file 1: Table S1.** Overview of the AIS per ISS-anatomical region, ISS, NISS of the presented polytrauma cases as allocated from an AAAM-certified specialist.

## Data Availability

The datasets used and/or analyzed during the current study are available from the corresponding author on reasonable request.

## Abbreviations

α: Krippendorff’s alpha (α) reliability coefficient
ΑΑΑΜ: Association for the Advancement of Automotive Medicine
AIS: Abbreviated Injury Scale
ATLS: Advanced Trauma Life Support
CI: Confidence interval
CT: Computed tomography
DGU: Deutsche Gesellschaft für Unfallchirurgie (German Trauma Society)
ICC: Intraclass correlation coefficient
IQR: Interquartile range
ISS: Injury Severity Score
MAIS: Maximum Abbreviated Injury Scale
NISS: New Injury Severity Score
RWTH: Rheinisch-Westfälischen Technischen Hochschule (University of Aachen)
