# Supplementary material for: Interobserver variability of injury severity assessment in polytrauma patients: does the anatomical region play a role?
Source: Eur J Med Res. 2021 Apr 15;26:35. doi: 10.1186/s40001-021-00506-w (PMC8051093; doi:10.1186/s40001-021-00506-w)
Supplement: Supplementary file 1 — Additional file 1: Table S1. Overview of the AIS per ISS-anatomical region, ISS, NISS of the presented polytrauma cases as allocated from an AAAM-certified specialist. [file 40001_2021_506_MOESM1_ESM.docx]

**Supplementary Table 1**

**Overview of the AIS per ISS-anatomical region, ISS, NISS of the presented polytrauma cases as allocated from an AAAM-certified specialist.**

| **Patient cases** | **Injuries of head and neck (AIS)** | **Injuries of face (AIS)** | **Injuries of thorax/thoracic spine (AIS)** | **Injuries of abdomen/visceral pelvis/lumbar spine (AIS)** | **Injuries of extremities/osseous pelvis/shoulder girdle (AIS)** | **Injuries of external skin/soft tissues (AIS)** | **ISS** | **NISS** |
| --- | --- | --- | --- | --- | --- | --- | --- | --- |
| **Patient 1** | Subdural hemorrhage 1,5cm (5)  Basal odontoid fracture of C2-vertebra (3) |  | Contusio cordis (1)  Unilateral lung contusion (2)  Unilateral tension pneumothorax (5) | Hepatic rupture 5x3cm (2)  Fracture of L2-vertebra with 22% loss of height (3) | Unstable pelvic fracture with bilateral involvement of the sacral lateral mass (4)  III^o^ open fracture of the right tibial pilon (3)  Closed fracture of the left tibial pilon (2) |  | **66** | **66** |
| **Patient 2** | Closed cranial vault fracture (2)  Closed fracture of Os sphenoidale (3)  Subarachnoidal hemorrhage (2)  Subdural hematoma 0,7cm (4)  Cerebral contusion 2,2x2cm (4)  Arch fracure of the C7-vertebra (2) | Closed fracture of Arcus zygomaticus (1) |  | Burst fracture of L1-vertebra with 25% loss of height (3) | Unilateral closed intraarticular fracture of the distal radius (2) |  | **29** | **41** |
| **Patient 3** | Subarachnoidal hemorrhage (2)  Bilateral subdural hematomas: right 0,9cm- left 0,8cm (4)  Atlantoaxial dislocation (3) |  | Multiple unilateral costal fracures: 9.-11. Costae (3) | Spleen laceration 3x1,2cm (2) | Unstable closed pelvic fracture with separation of the symphysis and involvement of the left dorsal Os ilium (3)  Closed bimalleolar fracture-dislocation of the left upper ankle joint (2) | Soft tissue lacera-tion of the scalp 9x0,7cm (1) | **34** | **34** |
| **Patient 4** | Subarachnoidal hemorrhage (2)  Open multifragmentary cranial vault fracture associated with dural tear, cranial base fracture and liquorrhea (4) |  | Multiple unilateral costal fracures: 4.-7. Costae (3)  Unilateral lung contusions (2) |  | Closed supracondylar fracure-dislocation of the left humerus (2) | Unilate-ral excoria-tion the thoracic wall 6x5cm (1) | **29** | **29** |
| **Patient 5** | Bilateral subdural hematomas 1,2cm (5) |  | Bilateral hematopneumo-thorax (3)  Pneumomediastinum (2)  Contusio cordis (1)  Unilateral unstable thorax with multiple costal fractures: 3.-8. Costae (3)  Traumatic spondylolisthesis T12-L1 with burst fracture of L1-vertebra and incomplete paraplegia (4)  Fracture of the Procc. spinosi T9-T12 (1) | Spleen laceration 1x1,5cm (2) | Closed S3-sacral fracture (2)  Closed medial fracture of the left clavicle (2)  Bilateral closed scapular fractures with unilateral involvement of the glenoid (2) |  | **45** | **50** |
| **Patient 6** |  |  | Burst fracture of T12-vertebra with 15% loss of height (2) |  | Unstable closed pelvic injury with symphysis separation and rightsided sacral fracture (4)  Dislocation of the right small toe in the metatarsophalan-geal joint (1)  Instability of the right ankle joint with ligament rupture and high-grade central cartilage defect of the tibial pilon (2) |  | **20** | **24** |
| **Patient 7** |  |  | Unilateral simple pneumothorax (2) | Retroperitoneal hematoma with unilateral transection of the internal iliac artery (4) | Unstable closed pelvic ring injury with fracture of the right pubic rami and unilateral transforaminal sacral fracture with contralateral sacroiliac joint separation (4)  Transverse fracture of the left acetabulum (2)  Closed transcondylar fracture of the left femur (3)  Weber type-C closed fracture-dislocation of the left upper ankle joint (2) | Occipital soft tissue lacera-tion 4x2cm (1) | **36** | **41** |
| **Patient 8** | Unilateral depressed closed cranial vault fracture (3)  Subarachnoidal hemorrhage (2)  Epidural hematoma 0,6cm (4)  Stable compression fracture of C6-vertebra (2) |  | Unilateral hematothorax 1500cc (4)  Unilateral multiple costal fractures: 1., 4. and 5. Costae (3)  Stable compression fracture of T8-vertebra (2) |  | Tossy type-III unilateral acromioclavicular joint separation (2) | Monocle hemato-ma (1) | **36** | **41** |
| **Patient 9** |  | Unilateral closed orbita floor fracture (2)  Unilateral closed maxillary wall fracture (2) | Unilateral tension pneumothorax (5)  Bilateral hematothorax 2050cc (4)  Bilateral multiple costal fractures with thoracic instability: left 3.-10. Costae, right 3.-5. and 8.-12. Costae (5)  Compression fractures of T4/T5/T7/T8-vertebrae with 10% loss of height (2) |  | Unilateral closed lateral clavicle fracture (2) | Excoria-tion of the thoracic wall 20x5cm (1)  Soft tissue lacera-tion of the right upper arm 7x3cm (1) | **33** | **66** |
| **Patient 10** |  |  | Bilateral lung contusions (3)  Unilateral simple pneumothorax (2)  Unilateral multiple costal fractures: 1.-3. Costae (3) | Spleen laceration 1 cm (2)  Transverse process fractures of the L1/L2/L3-vertebrae (2) | Bilateral transforaminal sacral fracture (4)  Closed fracture of the right femoral shaft (3)  Open multifragmentary fracture of the left femoral shaft (3) |  | **29** | **34** |

**Abbreviations:** AIS, Abbreviated Injury Scale; ISS, Injury Severity Score; NISS, New Injury Severity Score; AAAM, Association for the Advancement of Automotive Medicine.
